# Oral Health-Related Quality of Life among Children and Adolescents with Beckwith–Wiedemann Syndrome in Northern Italy

**DOI:** 10.3390/jcm11195685

**Published:** 2022-09-26

**Authors:** Patrizia Defabianis, Rossella Ninivaggi, Federica Romano

**Affiliations:** Department of Surgical Sciences, C.I.R. Dental School, University of Turin, 10126 Turin, Italy

**Keywords:** oral health-related quality of life, oral manifestations, macroglossia, rare diseases, Beckwith–Wiedemann syndrome, patient-related outcomes

## Abstract

Due to associated maxillofacial growth anomalies and the impairment of oral functions, macroglossia may negatively impact the oral health-related quality of life (OHRQoL) of people with Beckwith–Wiedemann syndrome (BWS). Therefore, the aim of this cross-sectional study was to determine the OHRQoL of Italian children and adolescents with BWS compared to healthy peers and to identify which symptoms related to macroglossia had the highest impact. A total of 48 patients with BWS and 48 age- and gender-matched controls completed the Italian version of OHIP-14 and a questionnaire on functional, oral and aesthetic outcomes. Parents of patients with BWS who had undergone tongue reduction surgery (TRS) answered additional questions related to surgery. The BWS group scored higher than controls on the total OHIP-14 and on the dimensions of oral function (*p:* 0.036) and psychosocial impact (*p:* 0.002), indicating a reduced OHRQoL. Neither gender nor age had an impact on OHRQoL. Scores were worse in children and adolescents treated with TRS, as most of them still had open bite malocclusion and speech difficulties. The OHRQoL of children and adolescents affected by BWS is worse than that of their healthy peers in spite of the surgical treatment of macroglossia.

## 1. Introduction

Beckwith–Wiedemann syndrome (BWS) is the most frequent genetic overgrowth disorder in infancy, with an incidence of 1 in 10,340 to 13,700 live births worldwide, affecting both males and females [1,2]. It is caused by abnormal methylation of one or both imprinted growth regulatory genes on chromosome 11p15 [2,3,4]. The clinical presentation is highly variable, but hallmark features are gigantism, exomphalos, hyperinsulinism and macroglossia [2,3]. Affected individuals are also have an increased likelihood of developing malignant tumours in early childhood [5]. 

Macroglossia is the predominant oral characteristic observed in BWS patients, resulting from muscle fibre hyperplasia and occurring in as many as 98% of patients [2,3,6]. According to Vogel et al. [7] its main signs and symptoms are dry and cracked tongue; difficulty chewing, swallowing and articulating sound; airway obstruction; and noisy breathing. As a consequence of the increased pressure of the tongue against and between the teeth, dentoskeletal disharmonies may develop, including mandibular prognathism, skeletal class III malocclusion, widened interdental spaces, anterior open bite and long facial height [8,9,10]. The clinical aspect, together with drooling and speech difficulties, could give the perception of mental retardation [11]. It has been also estimated that 48% of BWS patients suffer from sleep-disordered breathing, which could impair their skeletal growth pattern [12]. These functional and aesthetic implications may negatively affect body image, psychological well-being and social interactions in the daily living of affected subjects, thereby reducing their oral health-related quality of life (OHRQoL) [13,14,15]. Over time, the evolution of mandibular growth represents a health concern, even in adulthood [16].

OHRQoL is a specific aspect of QoL that has been recognized by the WHO as an important part of the Global Oral Health Program [17]. It has been defined as “the absence of negative impacts of oral conditions on social life and a positive sense of dentofacial self-confidence” [18]. It consists of a multidimensional construct that, according to Locker [19], is composed of three dimensions arranged hierarchically: the biological level (impairment), the behavioural level (discomfort, disability and functional limitation) and the social level (handicap). Despite the number of people affected by BWS worldwide, there is a gap in knowledge on OHRQoL in such a population. To date, only a few studies have been conducted on the effect of tongue reduction surgery (TRS) on taste function, oral malocclusion and speech intelligibility and articulation, with conflicting results [15,20,21]. 

Considering that traditional indicators can measure the entity of the physical disease but not its effects, especially during the childhood and adolescence life cycles, OHRQoL is an auxiliary tool that could be used to evaluate non-clinical aspects of health. Among the numerous measuring instruments, the validity and reliability of the Oral Health Impact Profile (OHIP) questionnaire has been widely documented [22]. The original version of the questionnaire was recently translated into multiple languages, providing reliable instruments to compare OHRQoL across populations. Its short 14-item version, the OHIP-14 [23], is preferred due its practicality, and its use has been documented among children with rare diseases [24,25,26]. It measures well-being according to functional, psychological and social domains that may be relevant in everyday life [23]. 

Therefore, the aim of this cross-sectional study was to assess the OHRQoL of Italian children and adolescents with BWS with respect to age- and gender-matched controls. 

A further aim was to determine which symptoms related to macroglossia had the highest impact. The null hypothesis is that there are no differences in OHRQoL between subjects with and without BWS. Identifying healthcare needs in patients diagnosed with BWS could contribute to the promotion of specific dental treatment guidelines and individualized psychological approaches to improve their well-being.

## 2. Materials and Methods

### 2.1. Study Participants, Recruitment and Setting

From January to May 2022, subjects with a confirmed diagnosis of BWS according to the 2018 criteria [3] were assessed for eligibility during initial dental examinations or routine dental visits at the Section of Paediatric Dentistry, Department of Surgical Sciences, University of Turin (Italy). Patients between 2 and 16 years of age of both genders and of any ethnicity were consecutively recruited. The exclusion criteria included subjects with other craniofacial malformations, patients older than 16 years and those with severe mental impairment or medical conditions that might influence taste perception and/or speech (such as cleft palate or hearing problems). 

Written informed consent was obtained from the parents/caregivers of all participants. The study was approved by the Institutional Ethical Committee of the “AOU Città della Salute e della Scienza” of Turin and was conducted according to the Declaration of Helsinki. Healthy controls matched for age and gender to BWS subjects were identified from the hospital database and were asked to participate in the study while attending routine appointments.

### 2.2. Data Collection and Questionnaires

Demographic and genetic data were extrapolated from medical records. (Epi)genetic alterations include loss of metalation at imprinting centre 2 (IC2-LoM), mosaic paternal uniparental isodisomy of chromosome 11 (UPD(11)pat), gain of methylation at imprinting centre 1 (IC1-GoM), maternally inherited inactivating mutations of cyclin-dependent kinase inhibitor 1C (CDKN1C), negative genotype and chromosomal rearrangement. 

A single trained interviewer administered the structured questionnaires face to face with participants, who answered the questions with assistance from their parents/caregivers. For children younger than 8 years, parents were asked to complete the questionnaires on their child’s behalf. OHRQoL was measured using the validated Italian version of OHIP-14 [27], which includes 14 items on mouth or tooth problems valued on a 5-point Likert scale from 0 to 4 (never = 0; hardly ever = 1; occasionally = 2; fairly often = 3; and very often = 4). The overall OHIP-14 score varies between 0 and 56; a higher value corresponds to a worse OHRQoL. Eight of the 14 items were assembled in four health areas, according to John et al. [28,29]: oral function (difficulty chewing and necessity to interrupt chewing), orofacial pain (toothache), orofacial appearance (self-consciousness of the oral condition) and psychosocial impact (dissatisfaction with respect to life and inability to function). These dimensions correspond to four of the seven subscales (physical disability, physical pain, psychological discomfort and handicap) into which OHIP-14 has been traditionally organized [23]. The score of each dimension, consisting of two questions, could range between 0 and 8.

The impact of macroglossia on oral, functional and aesthetic outcomes was analysed by administering a second questionnaire, which included questions extracted from the questionnaire formulated at the Great Ormond Street Hospital in London (GOSH questionnaire) and translated by the authors [30]. The presence (yes/no), the time (never, in the past or currently) and the frequency of a problem (from never to constantly) were rated for each question. 

Finally, parents of BWS children who had been surgically treated for macroglossia were asked about the surgical outcomes following TRS and their degree of satisfaction with TRS. Responses used a binary rating score (yes/no) and the grade of satisfaction was scored on a 0 to 10 VAS scale. 

### 2.3. Data Analysis

All data were anonymised, and each patient was identified by a code. Data were analysed using statistical software (SPSS, version 25, IBM Corp., Armonk, NY, USA) and published as aggregates. Qualitative data are presented as absolute and relative frequencies, and quantitative data are presented as mean and standard deviation (S.D.), median and range. The Fisher’s exact test and the chi-square test were used to evaluate any potential association between categorical variables. The Mann–Whitney U test and the Kruskal–Wallis test were applied to compare the differences in the quantitative variables between two or more groups, respectively. *p* values < 0.05 were considered statistically significant. 

## 3. Results

A total of 96 subjects (48 with BWS and 48 healthy individuals) were enrolled and completed the OHIP-14 and GOSH questionnaires. BWS Caucasian patients (28 females and 20 males) in the age range of 2–16 years (mean 9.0 ± 4.0 years) were included, and a homogenous matched population was sampled in terms of gender (28 females and 20 males) and age (mean 8.0 ± 3.4 years, *p*: 0.194). 

The BWS group included 23 (47.9%) individuals with the IC2-LoM genotype, 7 (14.6%) with the IC1-GoM genotype and 7 (14.6%) with the UPD(11)pat genotype, whereas 11 (22.9%) were genetically negative. A total of 38/96 (72.2%) patients presented with severe macroglossia, and 11/96 (22.9%) underwent TRS. About a half of the parents had been informed about the BWS diagnosis at the birth of their child (41.7%) or before leaving the maternity ward (8.3%), whereas the other half were informed later but before the child was three years old.

### 3.1. OHIP-14

Table 1 shows a comparison of the overall OHIP-14 and subscale scores for the BWS and control groups. Regarding the global questions, the mean score for OHRQoL was 4.7 ± 6.2 and 7.3 ± 8.2 for the control and BWS group, respectively, with a statistically significant difference (*p:* 0.022). Six patients with BWS (12.5%) scored 0 on all dimensions compared to 17 healthy controls (36.2%). The most severely affected domains were oral function (*p:* 0.036) and psychosocial impact (*p:* 0.002); on both these dimensions, the BWS group had higher scores than the control group. 

Considering the BWS subjects only, Table 2 shows an intragroup comparison of overall and subscale OHIP-14 scores according to gender, age, (epi)genotype and glossectomy. Children were divided into three groups according to their age: 2–5 years (preschoolers, 10 BWS), 6–11 years (schoolchildren, 25 BWS) and 12–16 years (adolescents, 13 BWS). Gender, age and (epi)genotype had no statistically significant effect on OHRQoL. Among patients who underwent TRS, the intervention had a negative impact on overall OHIP-14 (p: 0.001), as none of the operated patients has a score equal to zero; among untreated patients, six scored zero on all domains. The most affected areas were oral function, orofacial appearance and psychosocial impact (*p:* 0.003, *p:* 0.013 and *p:* 0.048, respectively). 

### 3.2. GOSH Questionnaire

Table 3 and Table 4 summarize oral, functional and aesthetic problems in both the BWS and control groups. Children with BWS experienced functional difficulties and oral problems more often than their healthy peers. A proportion of 45.8% of BWS patients had a currently an open bite, 10.4% had chewing difficulties and 22.9% suffered from snoring compared to 6.2%, 4.2% and 6.3% of controls, respectively. Drooling affected 27.1% of BWS patients, compared to 2.1% of their healthy peers. In particular, 60.0% of preschool children and 38.5% of adolescents with BWS were unable to control oral secretions.

As reported in Table 4, 39.6% of BWS patients participated in speech therapy, but 29.2% of them still had some problems with sound articulation. Seven patients expressed some concern for their tongue and three for their facial appearance. In about 30% of BWS children, the tongue protruded either occasionally or most of the time. 

Two-thirds of the parents/caregivers (70.8%) had been informed by medical staff about the possibility of speech difficulties as a result of an enlarged tongue; 82.4% of them had children 2 to 11 years old, and 17.6% had children older than 12 years. All parents of children with IC1-GoM and UPD(11)pat genotypes were aware, compared to 61% of parents of children with IC2-LoM and 54% of parents children with negative tests (*p*: 0.036). 

### 3.3. GOSH Questionnaire and Surgical Outcomes in BWS Patients with Glossectomy

A total of 11 BWS subjects (5 males and 6 females) with macroglossia underwent TRS at 20.1 ± 10.8 months of age (range 8–48 months). The same surgeon performed all the reductive surgeries using the keyhole technique. Ten subjects underwent a single surgical session; one subject required a second intervention. The majority of children with the IC1-GoM genotype (71.4%) were treated with TRS (*p*: 0.017). Parents/caregivers of children who did not undergo TRS declined surgery because they received opposing views or were advised against surgery by paediatric surgeons (45.0%). Only a minority of them (10.0%) was not informed about the surgical option. 

The impact of the tongue on oral cavity dimensions (Table 5), aesthetics and speech (Table 6) was statistically significantly different in BWS subjects who underwent TRS compared to those who did not; subjects treated with TRS were significantly more likely to report chewing difficulty (27.3% versus 5.4%, *p:* 0.028) and noisy breathing (45.5% versus 16.2%, *p:* 0.030). Additionally, 81.8% of surgically treated patients still had open bite posture, and 9.1% reported a history of open bite (*p:* 0.018).

As summarized in Table 6, whereas 54.5% of the surgically treated subjects still reported speech difficulties and 27.3% had experienced some problems in the past, most of the subjects who had not been treated with TRS did not report any speech problems (*p*: 0.002). The most disordered sounds were /r/, /t/ and /s/. Eight surgically treated children (72.7%) received speech therapy once a week for at least one year. They were also more concerned for their tongue appearance and function than non-surgically-treated children (*p*: 0.039).

Satisfaction with surgical outcomes was high for all parents, with the exception of two (81.8%), in terms of improvement in aesthetics, tongue mobility and sound articulation (8.6 ± 2.1, 9.0 ± 1.8 and 8.2 ± 3.2, respectively) to the point that they would opt for a surgical option again.

## 4. Discussion

This is the first observational study to examine OHRQoL, subjective oral health, speech and feeding performance in patients with BWS. Although only 48 participants were enrolled, the present findings indicate that all the investigated areas were negatively affected compared to age- and gender-matched healthy controls; thus, the null hypothesis was rejected. We administered the OHIP-14 questionnaire, which has been used to determine the OHRQoL of patients with other types of rare diseases affecting the oral cavity [24,25,26], as well as the GOSH questionnaire, which has been applied to patients with BWS to determine oral, functional and aesthetic concerns related to macroglossia [30]. 

We applied a four-dimensional characterization approach of perceived oral health instead of the traditional seven-dimension approach, as suggested by John et al. [28,29], who demonstrated through exploratory and confirmatory factor analysis using data from prosthodontics patients and general population subjects from six countries that a model with four health dimensions is sufficient to account for the OHIP’s latent structure [31,32]. The four identified OHRQoL areas represent the functional, pain-related, aesthetic and psychosocial impacts of oral disorders as perceived by individuals in their day-to-day lives [31,32].

The average OHIP-14 value of the study participants was 7.3 ± 8.2, which was higher than that of their peers (4.6), indicating relatively poorer oral health, on average, among the study participants. Due to the lack of published studies, it is not possible to compare these data with that of other studies on BWS; nonetheless, the present findings are in line with those reported for other rare diseases, such as Loeys–Dietz and Ehlers–Danlos syndromes, which are accompanied by associated dental and craniofacial anomalies [25,33]. 

Oral function and psychosocial impact were the most affected OHIP-14 health domains among BWS patients. As a consequence of macroglossia, BWS patients often develop orofacial growth disturbances and experience impaired oral functions, such as difficulties in feeding, chewing and sound articulation [8,9,10]. Moreover, tongue enlargement could also have aesthetic implications because open mouth, together with tongue protrusion and drooling, may be perceived as an intellectual disability. This condition may cause significant difficulties in everyday life in BWS subjects, reducing their overall OHRQoL [11,13,14,15]. 

Interestingly, gender, age and (epi)genotype did not impact either on the overall or individual OHIP-14 subscale scores. In contrast, BWS patients who had undergone TRS still had a more negative attitude with respect to their oral function and facial appearance; in addition, they seemed to be more conscious of the difficulties related to the disease. Generally speaking, it has been estimated that more than 40% of BWS paediatric patients require TRS to prevent or to correct the functional and aesthetic consequences of their enlarged tongue [14]. In our sample, about two-thirds of BWS patients exhibited macroglossia and anterior open bite, but only 23% of them had undergone TRS. Recent papers call into question the benefits of TRS in controlling open bite, mandibular growth rate and skeletal III malocclusion [10,20].

Because the OHIP-14 does not specifically ask which oral manifestation is associated with OHRQoL health domains, we complemented the OHIP-14 results with obtained through the GOSH questionnaire. Drooling, feeding and articulating sound were the functional areas most frequently reported to be impaired. Brodsky [34] reported that drooling (i.e., spilling of saliva from the mouth onto the lips, chin, neck and clothing) is a physiological finding in infants and young children, particularly when they are learning a new motor skill or during the eruption of a new tooth; however, drooling is no longer considered normal after 3 or 4 years of age. In line with the findings reported by Shipster et al. [30], 27.1% of BWS patients enrolled in this study reported constant drooling, and 20.8% had experienced it during infancy. According to age-stratified data, 60.0% of preschool children currently drooled, and 38.5% of adolescents were unable to control oral secretions. Drooling often results in oral and perioral infections, irritated lower-third facial skin and dehydration, and drooling has been reported to improve following TRS when lip closure can be achieved [30,35]. Due to the perception of mental retardation, subjects who drool may feel a sense of isolation and rejection by their peers, which could strongly affect their OHRQoL [30,36].

Consistent with the present findings on OHIP-14, speech difficulty was a frequent issue for BWS patients, negatively influencing their daily life. Although 39.6% of BWS patients had participated in speech therapy, 29.2% of them still reported problems with sound articulation. Speech errors involve mostly the interdental /s/; the addental /t/ and /d/ [37,38]; and the labiodental /r/, /f/ and /v/ sounds [15,39]. Due to the high occurrence of such problems, parents should be promptly advised of a BWS diagnosis. In this study, about 70% of parents had been informed about these problems at the moment their child was born, compared to the 40% reported by Van Borsel et al. [39]. Considering that 82% of children in the present study were younger 11 years old and 18% children were older than 12, it seems reasonable to conclude that diagnostic counselling has become increasingly more precise over the last 10 years. All parents of IC1-GoM and UPD(11)pat children were informed about possible phonation difficulties, probably because these molecular alterations are related to more pronounced tongue enlargement [2].

BWS children with macroglossia also have feeding issues involving problems with bolus manipulation, difficulty in controlling liquids and anterior bolus loss, resulting in increased risk of aspiration [38]. Prandeville et al. [38] and Maas et al. [15] stated that these feeding difficulties are due to the movements of the enlarged protruding tongue during the cohesive bolus formation and food propulsion to the back of the oral cavity. In the present study, only 10% of patients complained about this issue; in particular, one child was unable to hold a bolus in the oral cavity. It may be argued that difficulties are mainly related to dental malocclusion and craniofacial anomalies, which affect chewing ability [8]. 

In relation to TRS outcomes, several studies have reported positive effects of glossectomy from the functional and aesthetic perspectives, although some authors highlight the possibility of hypoglossal nerve injury with consequent impairment of speech and chewing performance [30,35]. In the present study, 27.3% of BWS patients who underwent TRS had chewing difficulty due to limitation of tongue mobility compared to 5.4% of patients who had not been surgically treated. Moreover, one subject who had undergone two tongue reduction surgeries had marked limitations in tongue tip mobility and elevation and was unable to touch the labial surfaces of any teeth. These findings are in line with those reported by Tomlinson, who found that three of eleven patients who had undergone TRS had marked limitations of tongue mobility [37]. The percentages of TRS-treated patients with residual speech errors and open bite was 54.5% and 81.8%, respectively, calling into question the utility of performing TRS to correct or to avoid craniofacial deformities [10,20].

In relation to the aesthetic outcomes, only 14.6% of children/adolescents with BWS were concerned about the appearance of their tongue. Moreover, most of the parents reported positive ratings of the surgery and stated that they would opt for surgery again because TRS improved their children’s facial appearance, as the tongue was no longer protruding, in addition to reducing some functional difficulties [15,30]. Shipster et al. reported that parents are mostly concerned about judgment or unpleasant questions about their children’s learning difficulties due to their facial appearance [30]. Treated children had worse scores on the overall OHIP-14 and the orofacial appearance dimension compared to their untreated counterparts. We speculate that the treatment protocol is insufficient to completely address the adverse oral health impact on the patients. However, we did not collect data on preoperative ratings. 

Limitations of this study include the small sample size, although BWS is considered a rare disease, as well as the enrolment of a convenience sample of Caucasian children/adolescents diagnosed with BWS. Thus, the present findings may not be generalizable to all people affected by BWS. Another limitation is that participants were assisted by parents/caregivers in responding to the questionnaires, especially for questions regarding their first years of life, which they were unable to answer independently. The risk is that children’s personal reports may not fully detect patients’ quality of life, as some answers may have been underestimated because parents/caregivers did not accurately remember the events or possible inconveniences experienced by their children at that time [40]. Finally, as the oral conditions of the participants were not examined, the subjective assessment of the OHRQoL could not be verified. 

## 5. Conclusions

This cross-sectional study addresses a gap in knowledge of a rare disease and provides insight on the OHRQoL status, subjective oral health assessment, speech and feeding performance of children and adolescents with BWS. The OHIP-14 scores of children with BWS were higher compared to those of their healthy peers, which were associated with a reduced OHRQoL. Gender, age and (epi)genotype did not have an impact on OHRQoL, whereas TRS did. Children and adolescents treated with TRS had had lower scores than untreated children, as most of them still had open bite malocclusion and some residual speech difficulties. Considering the problems affecting young people with BWS are, both they and their parents/caregivers should receive additional support from the healthcare system.

## Figures and Tables

**Table 1:** Overall and subscale OHIP-14 scores by group.

OHIP-14 Domain (Maximum Possible Score)	Group				*p* Value
	BWS Patients (*N* = 48)		Controls (*N* = 48)		
	Mean ± SD	Median (min/max)	Mean ± SD	Median (min/max)	
Overall OHIP (56)	7.3 ± 8.2	5.5 (0/37)	4.7 ± 6.2	2.0 (0/23)	0.022
Oral function (8)	1.0 ± 1.4	0.0 (0/5)	0.6 ± 1.2	0.0 (0/5)	0.036
Orofacial pain (8)	1.8 ± 1.8	1.0 (0/6)	1.4 ± 1.7	0.5 (0/5)	0.191
Orofacial appearance (8)	0.6 ± 1.4	0.0 (0/6)	0.5 ± 1.1	0.0 (0/5)	0.791
Psychosocial impact (8)	1.1 ± 1.8	0.0 (0/8)	0.2 ± 0.7	0.0 (0/3)	0.002

**Table 2:** Overall and subscale OHIP-14 scores in BWS patients by gender, age, genotype and tongue reduction surgery (mean ± SD, median, (range)).

Variable	OHIP-14 Scores				
	Overall	Oral Function	Orofacial Pain	Orofacial Appearance	Psychosocial Impact
Gender					
Female (*N* = 28)	8.9 ± 9.9 6.0 (0/37)	1.1 ± 1.5 0.0 (0/5)	2.2 ± 2.1 2.0 (0/6)	0.9 ± 1.6 0.0 (0/6)	1.2 ± 2.1 0.0 (0/8)
Male (*N* = 20)	4.9 ± 3.9 3.5 (0/13)	0.9 ± 1.2 0.0 (0/4)	1.2 ± 1.3 0.0 (0/4)	0.2 ± 0.5 0.0 (0/2)	0.8 ± 1.2 0.0 (0/4)
*p* Value	0.395	0.714	0.130	0.116	0.807
Age group					
2–5 years (*N* = 10)	2.8 ± 2.7 2.0 (0/8)	0.6 ± 0.8 0.0 (0/2)	0.9 ± 1.6 0.0 (0/5)	0.0 ± 0.0 0.0 (0)	0.5 ± 0.5 0.5 (0/1)
6–11 years (*N* = 25)	9.0 ± 8.5 8.0 (0/35)	1.3 ± 1.7 0.0 (0/5)	2.2 ± 1.9 2.0 (0/6)	0.8 ± 1.4 0.0 (0/5)	1.1 ± 1.8 0.0 (0/6)
12–16 years (*N* = 13)	7.4 ± 9.3 6.0 (1/37)	0.9 ± 1.0 1.0 (0/2)	1.9 ± 1.7 1.0 (0/6)	0.7 ± 1.7 0.0 (0/6)	1.4 ± 2.5 0.0 (0/8)
*p* Value	0.057	0.685	0.116	0.095	0.933
(Epi) genotype					
IC2-LoM (*N* = 23)	6.7 ± 8.4 4.0 (0/35)	1.0 ± 1.5 0.0 (0/5)	1.7 ± 1.9 1.0 (0/6)	0.7 ± 1.4 0.0 (0/5)	0.7 ± 1.2 0.0 (0/5)
Negative (*N* = 11)	8.7 ± 9.9 6.0 (1/37)	1.1 ± 11 1.0 (0/3)	2.3 ± 1.9 2.0 (0/6)	0.7 ± 1.8 0.0 (0/6)	1.3 ± 2.5 0.0 (0/8)
IC1-GoM (*N* = 7)	10.0 ± 7.1 8.0 (2/21)	1.4 ± 1.5 1.0 (0/4)	2.1 ± 1.8 2.0 (0/5)	1.0 ± 1.60 1.0 (0/2)	2.5 ± 2.3 1.0 (0/6)
UPD(11)pat (*N* = 7)	4.0 ± 4.8 2.0 (0/13)	0.7 ± 1.2 0.0 (0/3)	1.3 ± 1.6 1.0 (0/4)	0.0 ± 0.0 0.0 (0/0	0.3 ± 0.5 0.0 (0/1)
*p* Value	0.171	0.604	0.551	0.141	0.075
TRS					
No (*N* = 37)	6.0 ± 8.4 3.0 (0/37)	0.7 ± 1.2 0.0 (0/5)	1.7 ± 1.8 1.0 (0/6)	0.5 ± 1.3 0.0 (0/6)	0.8 ± 1.6 0.0 (0/8)
Yes (*N* = 11)	11.5 ± 6.0 10.0 (2/21)	2.1 ± 1.4 2.0 (0/4)	2.4 ± 2.0 2.0 (0/6)	1.2 ± 1.3 1.0 (0/4)	2.1 ± 2.3 1.0 (0/6)
*p* Value	0.001	0.003	0.263	0.013	0.048

IC1-GoM, gain of methylation at imprinting centre 1; IC2-LoM, loss of methylation at imprinting centre 2; UPD(11)pat, mosaic paternal uniparental isodisomy of chromosome 11; S.D., standard deviation; TRS, tongue reduction surgery.

**Table 3:** Oral and related functional aspects by group.

Variable	Group		*p* Value
	BWS Patients (*N* = 48)	Controls (*N* = 48)	
Wide interdental spaces (n, %)			0.627
Never	32 (50.0)	32 (50.0)	
Currently	13 (46.4)	15 (53.6)	
In the past	3 (75.0)	1 (25.0)	
Open bite (n, %)			<0.001
Never	21 (33.3)	42 (66.7)	
Currently	22 (88.0)	3 (12.0)	
In the past	5 (62.5)	3 (37.5)	
Tongue ulcerative lesions (n, %)			1.000
Never	46 (50.0)	46 (50.0)	
Currently	2 (66.7)	1 (33.3)	
In the past	0 (0.0)	1 (100.0)	
Chewing difficulties (n, %)			0.016
Never	32 (43.2)	42 (56.8)	
Currently	5 (55.6)	4 (44.4)	
In the past	11 (84.9)	2 (15.4)	
Airway obstruction (n, %)			0.002
Never	34 (42.5)	46 (57.5)	
Currently	4 (100.0)	0 (0.0)	
In the past	10 (83.3)	2 (16.7)	
Snoring (n, %)			0.010
Never	29 (40.8)	42 (59.2)	
Currently	11 (78.6)	3 (21.4)	
In the past	8 (72.7)	3 (27.3)	
Drooling (n, %)			<0.001
Never	25 (36.8)	43 (63.2)	
Currently	13 (92.9)	1 (7.1)	
In the past	10 (71.4)	4 (28.6)	

**Table 4:** Aesthetics, feeding and speech problems by group.

Variable	Group		*p* Value
	BWS patients (*N* = 48)	Controls (*N* = 48)	
Tongue protrusion (n, %)			<0.001
Never	34 (41.5)	48 (58.5)	
Sometimes	12 (100.0)	0 (0.0)	
Constantly	2 (100.0)	0 (0.0)	
Concern about the tongue (n,%)			0.012
No	41 (46.1)	48 (53.9)	
Yes	7 (100.0)	0 (0.0)	
Concern about facial appearance (n, %)			0.671
No	45 (49.5)	46 (50.5)	
Yes	3 (60.0)	2 (40.0)	
Feeding difficulties (n, %)			0.714
No	43 (48.9)	45 (51.1)	
Yes	5 (62.5)	3 (37.5)	
Speech problems (n, %)			0.011
Never	29 (40.8)	42 (59.2)	
Currently	14 (77.8)	4 (22.2)	
In the past	5 (71.4)	2 (28.6)	
Speech therapy (n, %)			0.003
No	29 (40.8)	42 (59.2)	
Yes	19 (76.0)	6 (24.0)	

**Table 5:** Oral and related functional aspects according to tongue reduction surgery (TRS).

Variable	Group		*p* Value
	No TRS (*N* = 37)	TRS (*N* = 11)	
Wide interdental spaces (n, %)			0.630
Never	25 (78.1)	7 (21.9)	
Currently	9 (69.2)	4 (30.8)	
In the past	3 (100.0)	0 (0.0)	
Open bite (n, %)			0.018
Never	20 (95.2)	1 (4.8)	
Currently	13 (59.1)	9 (40.9)	
In the past	4 (80.0)	1 (20.0)	
Tongue ulcerative lesions (n, %)			0.410
Never	36 (78.3)	10 (21.7)	
Currently	1 (50.0)	1 (50.0)	
In the past	0 (0.0)	0 (0.0)	
Chewing difficulties (n, %)			0.028
Never	28 (87.5)	4 (12.5)	
Currently	2 (40.0)	3 (60.0)	
In the past	7 (63.6)	4 (36.4)	
Airway obstruction (n, %)			0.054
Never	29 (85.3)	5 (14.7)	
Currently	3 (75.0)	1 (25.0)	
In the past	5 (50.0)	5 (50.0)	
Snoring (n, %)			0.030
Never	26 (89.7)	3 (10.3)	
Currently	6 (54.5)	5 (45.5)	
In the past	5 (62.5)	3 (37.5)	
Drooling (n, %)			0.478
Never	21 (84.0)	4 (16.0)	
Currently	9 (69.2)	4 (30.8)	
In the past	7 (70.0)	3 (30.0)	

**Table 6:** Aesthetic, feeding and speech problems after tongue reduction surgery (TRS).

Variable	Group		*p* Value
	No TRS (*N* = 37)	TRS (*N* = 11)	
Tongue protrusion (n, %)			0.552
Never	26 (76.5)	8 (23.5)	
Sometimes	10 (83.3)	2 (16.7)	
Constantly	1 (50.0)	1 (50.0)	
Concern about the tongue (n, %)			0.039
No	34 (82.9)	7 (17.1)	
Yes	3 (42.9)	4 (57.1)	
Concern about facial appearance (n, %)			1.000
No	35 (77.8)	10 (22.2)	
Yes	2 (66.7)	1 (33.3)	
Feeding difficulties (n, %)			0.321
No	34 (79.1)	9 (20.9)	
Yes	3 (60.0)	2 (40.0)	
Speech problems (n, %)			0.002
Never	27 (93.1)	2 (6.9)	
Currently	8 (57.1)	6 (42.9)	
In the past	2 (40.0)	3 (60.0)	
Speech therapy (n, %)			0.016
No	26 (89.7)	3 (10.3)	
Yes	11 (57.9)	8 (42.9)	

## Data Availability

The data underlying this article cannot be shared publicly due to the vulnerable study population. Data will be shared upon reasonable request to the corresponding author.
